# Norcaradiene–Cycloheptatriene Equilibrium: A Heavy-Atom Quantum Tunneling Case

**DOI:** 10.1021/acs.joc.4c00464

**Published:** 2024-06-18

**Authors:** Juan García de la Concepción, José C. Corchado, Pedro Cintas, Reyes Babiano

**Affiliations:** † Departamento de Química Orgánica e Inorgánica, Facultad de Ciencias, and IACYS-Green Chemistry and Sustainable Development Unit, Universidad de Extremadura, 06006 Badajoz, Spain; ‡ Departamento de Ingeniería Química y Química Física, Facultad de Ciencias, and ICCAEx, Universidad Extremadura, 06006 Badajoz, Spain

## Abstract

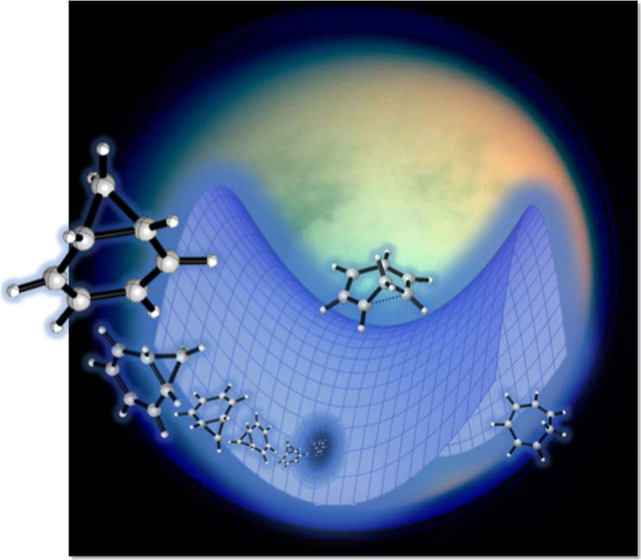

The equilibrium between norcaradiene and cycloheptatriene, which has captivated chemists for more than half a century, is revisited by state-of-the-art quantum chemical calculations. Our theoretical data significantly deviate from the experimental results (*J*. *Am*. *Chem*. *Soc*.*,***1981,***26,* 7791–7792), especially at low temperatures, where isomerization is dominated by heavy-atom tunneling. This effect results in an extremely short half-life for norcaradiene, rendering it undetectable. This work sheds light on this equilibrium, updating the kinetic and thermodynamic data while also expanding the repertoire of organic reactions controlled by this exotic quantum effect.

## Introduction and Background

**Scheme 1:**
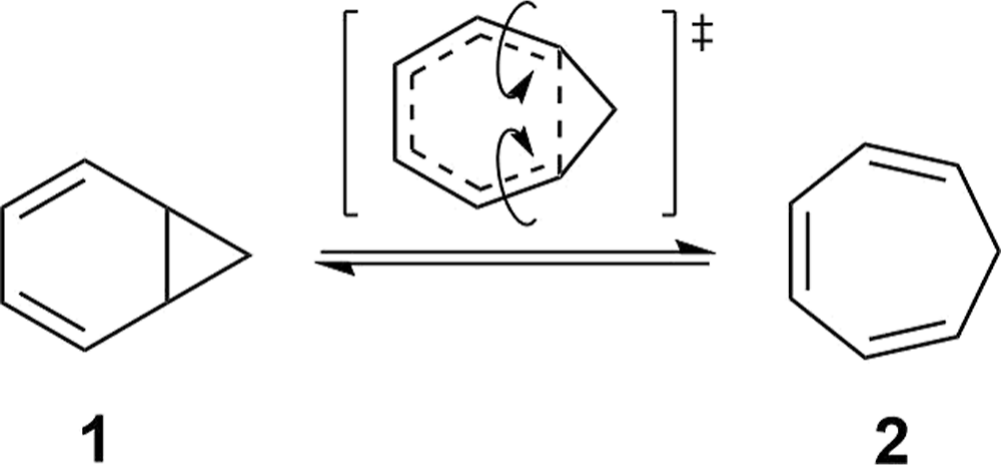
Norcaradiene–Cycloheptatriene Isomerization

The norcaradiene(**1**)–cycloheptatriene(**2**) equilibrium involves an orbital-symmetry allowed pericyclic reaction,^1^ which still raises questions despite a long odyssey of experiment and theory aimed at identifying the norcaradiene. This disrotatory electrocyclic reaction (Scheme 1) has garnered the interest of chemists for more than half a century.^2−4^

**Scheme 2:**
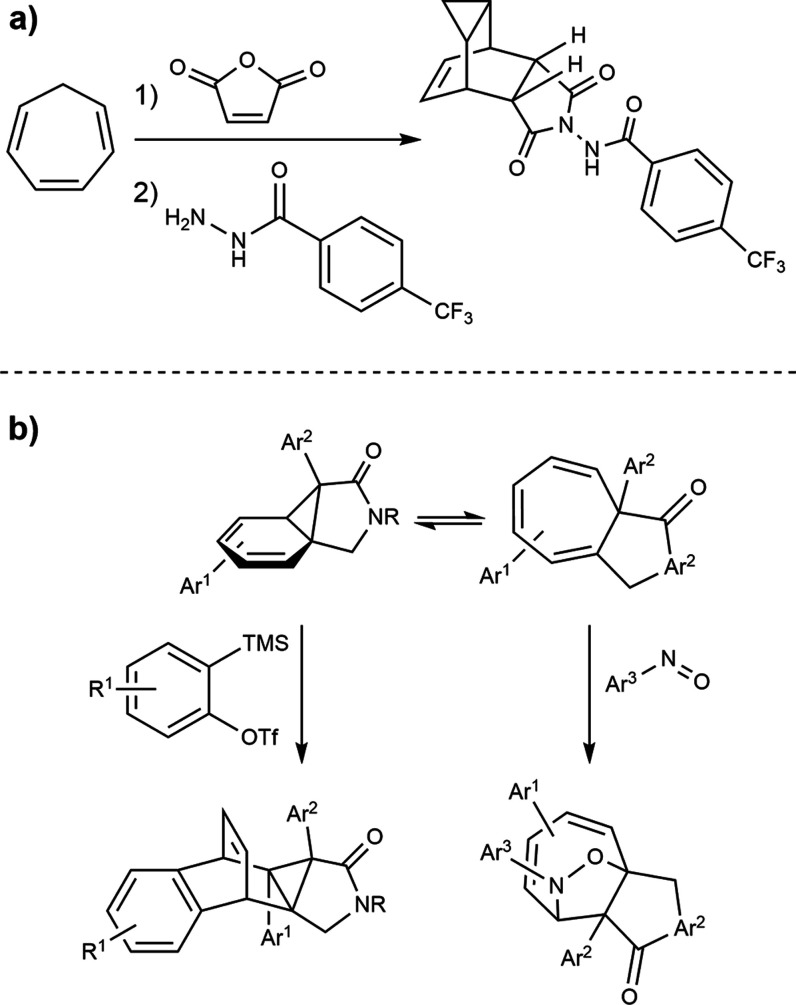
Synthetically Useful Cycloadditions Exploiting the Norcaradiene–Cycloheptatriene Equilibrium: (a) Towards the Antiviral Drug Tecovirimat and (b) Valence-Isomer Selective Cycloadditions

Practical applications of this equilibrium usually involve cycloaddition reactions as is illustrated by the recent synthesis of Tecovirimat, an antiviral drug for treating monkeypox.^3^ The key strategy involves a one-step Diels–Alder reaction intercepting in situ-generated norcaradiene (Scheme 2a). More recently, a series of valence-isomer selective cycloaddition reactions of cycloheptatriene-norcaradiene have been disclosed,^4^ namely the anomalous thermally forbidden suprafacial [6 + 2]-cycloadditions with nitroso compounds and norcaradiene-selective [4 + 2]-cycloadditions employing benzynes (Scheme 2b).

**Chart 1:**
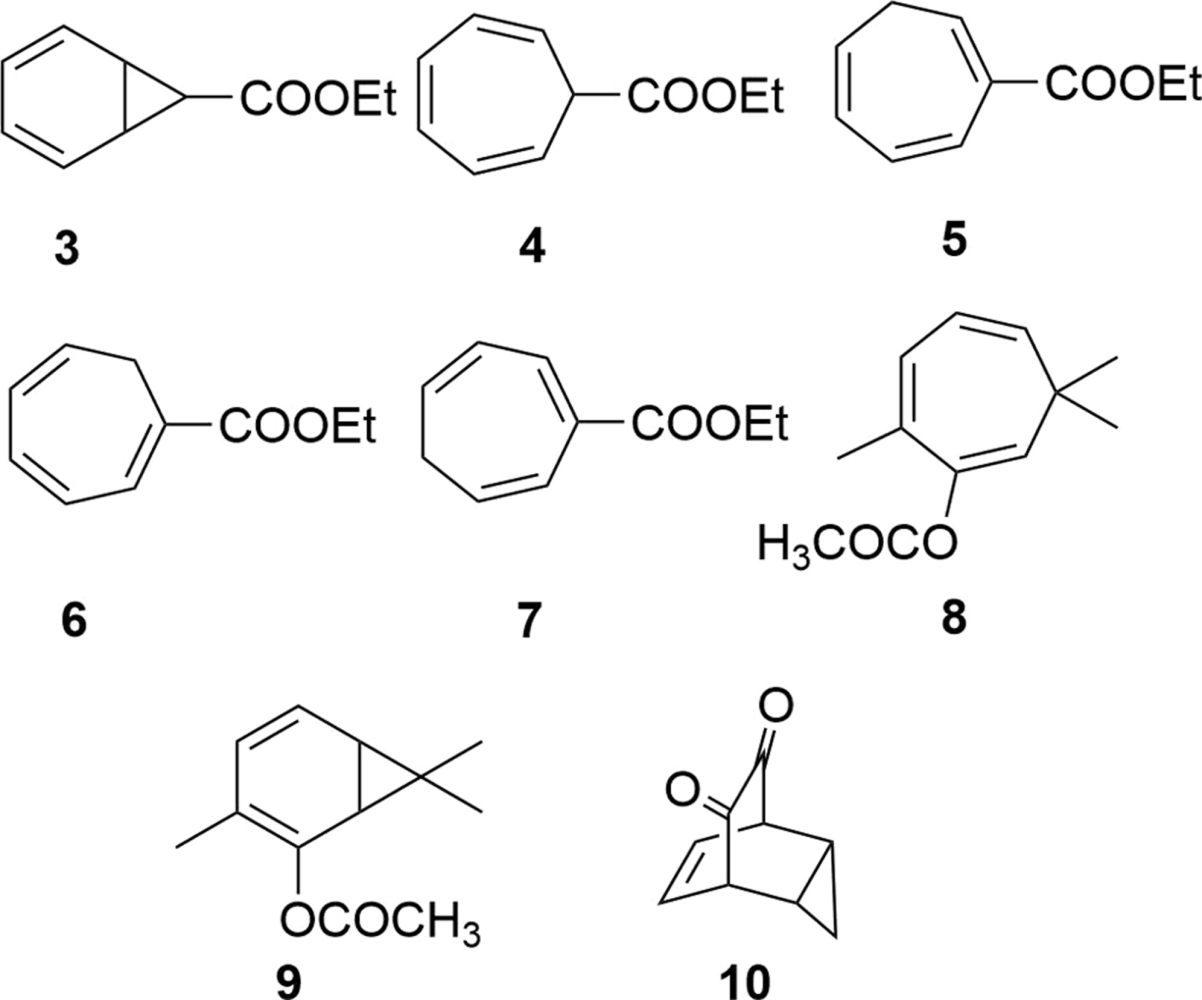
Key Structures Considered along the Chemical History of the Norcaradiene–Cycloheptatriene Equilibrium

**Chart 2:**
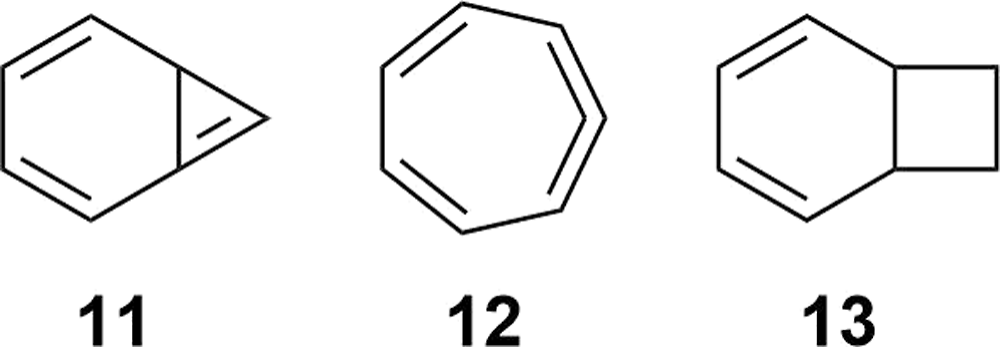
Structure of Bicyclo[4.1.0]hepta-2,4,6-triene (**11**), Cyclohepta-1,2,4,6-tetraene (**12**) and Bicyclo[4.2.0]octa-2,4-diene (**13**)

The study of the interconversion between these species began as early as 1885 when Buchner and Curtius postulated that decomposition of 3-diazo-2-ethoxyprop-1-ene in benzene led to ethyl-7-norcaradienecarboxylate (3, Chart 1).^5^ The debate on the elusive equilibrium was launched by Meerwein, who in 1957 described the above product as a mixture of **3** and cycloheptatriene derivative **4**.^2^ Notably, one year before, Doering et al. had proposed the product to be a mixture of trienes **5**–**7**, thereby implying the existence of cycloheptatriene **4**.^6^ Corey and co-workers managed to detect both species using NMR spectroscopy, studying the equilibrium between the natural product eucarbone **8** and its isomer **9** (Chart 1).^7^ Up to then, substituted species featuring both norcaradiene and cycloheptatriene backbone had been reported, albeit not the naked hydrocarbons, nevertheless.^8^ Certainly, Corey’s pioneering work motivated further pursuits to detect norcaradiene by spectroscopic methods, which were unsuccessful.^9−11^ It was not until 1981 that Rubin documented the first experimental detection of norcaradiene using UV/vis spectroscopy by photodissociation of tricyclo[3.2.2.0]non-6-ene-8,9-dione (**10**) at 77 K.^12^ This result would suggest that at cryogenic temperatures, the elusive species survived long enough to record a UV spectrum. Since the only “experimental detection” of norcaradiene arises from the seminal study by Rubin, the corresponding kinetic data reported have repeatedly been used as a reference for norcaradiene-to-cycloheptatriene isomerization.^12^ In addition, the reverse cycloheptatriene to norcaradiene ring closure was based on a previous assumption that the ratio between both isomers should be 0.001 at 293.15 K, if one accepts that norcaradiene exhibits the same diene character as bicyclo[4.2.0]octa-2,4-diene (**13**, Chart 2, for further details see Supporting Information),^13^ a figure taken as thermodynamic reference for the isomeric population. Even though the attention to this intriguing transformation has grown steadily throughout the last years,^14^ a satisfactory rationale has not yet been established.

On the findings of such investigations, the detection of the two isomers in equilibrium has been relatively easy to achieve for some derivatives, as substitution at certain ring positions decisively modifies the equilibrium. The most common derivatizations involve either mono- or disubstitution of the hydrogen atoms of the methylene group and other C–H bonds with π-acceptor groups. Additionally, modifications to amplify mesomeric effects at diverse exocyclic positions are typically employed.^15−19^ The stabilizing effect of norcaradiene in the presence of a strong electron-withdrawing group (EWG) at the methylene position was explained by Hoffmann using a substituted cyclopropane as analog.^20^ These π-acceptor groups induce a decrease in energy of the lowest unoccupied molecular orbital that efficiently interacts with the occupied highest occupied molecular orbital (HOMO) orbital within the cyclopropane ring. Consequently, the electrons of the HOMO become delocalized across the adjacent EWG, yielding a redistribution of electron density away from the cyclopropane. This charge redistribution weakens the antibonding character of the bridgehead carbons, resulting in shortening, whereas the vicinal σ bonds experience lengthening and leading to stabilization of the norcaradiene structure. Overall, unsaturated bicyclic three-membered ring-bearing molecules which isomerize throughout ring expansion reactions are still eliciting considerable interest, as exemplified by other alkyl-substituted bicyclic precursors such as azulenones^21^ and heterocyclic systems, namely oxepines,^22^ azepines,^23^ and benzazirines.^24,25^ These heterocycles have shown that the half-life of the bicyclic intermediate is exceedingly low because it undergoes isomerization owing to quantum mechanical tunneling (QMT).

QMT is an effect that allows chemical reactions to occur at energies below their barriers.^26−32^ This effect becomes largely relevant in astrochemistry, thereby enabling the reaction to occur in low-temperature environments (see for instance^33,34^). Light atom tunneling has been detected in processes such as enzymatic catalysis, abstraction reactions, organometallic transformation, and reactions involving carbenes, among others.^35−38^ QMT effects involving heavier atoms are termed heavy-atom tunneling. These processes, even if regarded as exotic and being nontrivial to be detected,^39^ play a crucial role in the isomerization of organic molecules, such as cycloaromatizations, pericyclic reactions, radical rearrangements, and π-bond-shifting.^40,41^ While these rearrangements primarily involve carbon atoms, examples of heavy-atom tunneling with even heavier atoms have also been documented.^42^ In some instances, QMT prevents the detection of molecules due to its extremely rapid decay. This phenomenon, known as “tunneling instability”, has recently been observed in systems distinct from those mentioned so far, such as hexazine, pentazine, and diazo compounds.^43,44^ However, this effect has likewise been observed for molecules similar to norcaradiene, such as bicyclo[4.1.0]hepta-2,4,6-triene (**11**).^45^ The detection of this intermediate proved to be unattainable due to its swift conversion into its isomer **12**, driven by heavy-atom tunneling. A potential and close relationship between this system and that of norcaradiene is that fluorination at positions C1 and C5 of **11** is required to decelerate the rearrangement. Then, the elusive nature of norcaradiene may be a result of its isomerization being controlled by QMT.

Since the early 1970s^46^ to the present day,^32,39,40^ numerous cases of heavy-atom tunneling have been identified. Other cases, however, may have gone unnoticed, as the present study unveils. Herein, we have tried to shed light on mechanistic understanding of the norcaradiene–cycloheptatriene isomerization, aided by state-of-the-art QM calculations. Our results show noticeable discrepancies among experimental data and misconceptions on thermochemical analysis. The study provides compelling computational evidence that this isomerization is ultimately controlled by QMT, preventing the alleged detection of norcaradiene at low temperatures,^12^ similar to the case found for fluorinated bicycloheptatriene.^45^ Our analysis accounts for an accurate reinterpretation of data collected so far and expands the repertoire of molecular isomerizations where tunneling should reasonably be involved.

## Results and Discussion

Evaluation of kinetic and thermodynamic data for the equilibrium between norcaradiene (**1**) and cycloheptatriene (**2**) has been carried out with the double hybrid revDSD-PBEP86^47,48^ in combination with the D3BJ empirical dispersion correction.^49,50^ Geometry optimizations were carried out with the correlation consistent Dunning basis set jun-cc-pVTZ^51−53^ with energy refinements performed by extrapolating to the complete basis set limit.^54−56^ Validation of this theoretical framework was accomplished by comparing the revDSD-PBEP86(D3BJ)/CBS energies with those obtained by means of CCSD(T)-F12^57,58^ in combination with the cc-pVTZ-F12 basis set and leading to a good concordance (see Supporting Information for full computational details). Even though previous computational studies for this system have been carried out with CASSCF and MROPT2 calculations, it is noteworthy that our CCSD(T)-F12 energy corrections on revDSD geometries show a *T*_1_ diagnostic of 0.01 for the three stationary points; therefore, no multiconfigurational character should be expected. Hereafter, all energy results are given at the above-mentioned level of theory including solvation effects with the SMD method^59,60^ in cyclohexane (referred to as revDSD/CBS from now onward). Kinetic calculations were carried out within the canonical variational transition state theory (CVT) with the small curvature tunneling approximation (SCT).^61^ Density functional theory (DFT), coupled cluster and kinetic calculations were carried out with the Gaussian 16, Orca 5, and Pilgrim software packages, respectively.^62−65^ The full quantum chemical and kinetics calculations can be found in Supporting Information.

The revDSD level of theory provides a geometry of the cycloheptatriene very close to that obtained through electron diffraction data,^66^ as inferred from Table S1. The approximate experimental results give a free energy difference between the two isomers on the order of 4 kcal mol^–1^ at 293 K.^12,13^ It is crucial to emphasize that the approximations used to derive the energy difference between the isomers rely on experimental data, whose details are not explicitly mentioned. Accordingly, the only data that can be harnessed for comparative purposes are theoretical. Previous calculations carried out with B3LYP, MROPT2, and CASSCF in the gas phase give electronic energy differences of 6.4, 7.3, and 11.8 kcal mol^–1^ respectively.^67^ On the other hand, the use of the hybrid functional ωB97X-D gives free energy differences at 298.15 K of 4.1 kcal mol^–1^ in chlorobenzene and 2.7 kcal mol^–1^ in xylene and acetone.^14^ The present results, obtained with revDSD/CBS and with the inclusion of anharmonic zero point energy (ZPE_Anh_) corrections computed with the vibrational perturbative theory to second-order VPT2,^68^ provide a free energy difference at 298.15 K of 5.2 kcal mol^–1^. The CCSD(T)-F12/cc-pVTZ-F12 calculations with the aforementioned corrections give a free energy difference of 6.1 kcal mol^–1^. Considering that the experimental approximations are based on the activity of a molecule other than norcaradiene, it is likely that the population of norcaradiene in a nonpolar environment at 298.15 K is considerably lower than that reported experimentally (0.001), being ≤0.0002 and ≤0.00003 for the revDSD/CBS and CCSD(T)-F12/cc-pVTZ-F12, respectively.

**Figure 1:**
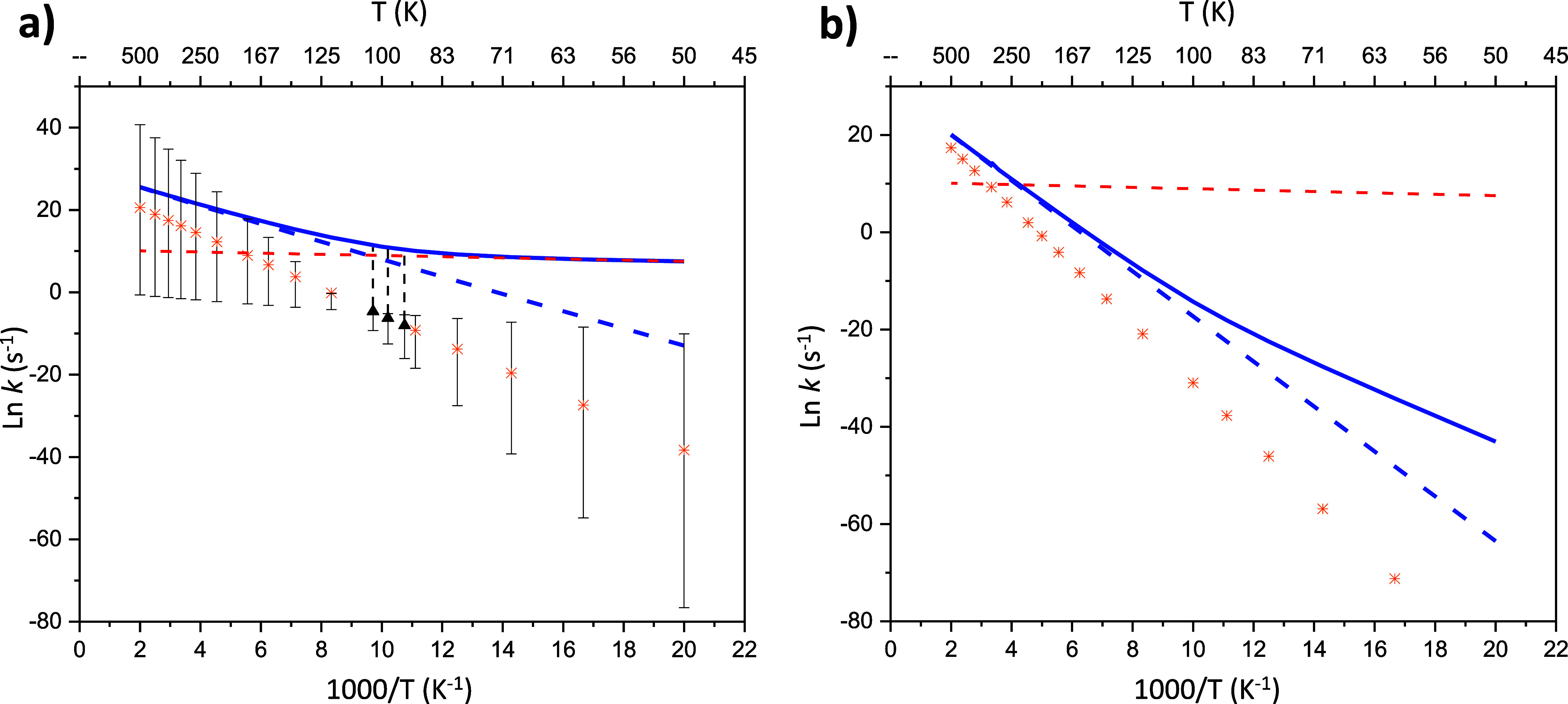
Rate constants for the isomerization between norcaradiene and cycloheptatriene. The solid blue lines represent calculations using the CVT/SCT theory, while the dashed blue lines correspond to calculations with the CVT theory. The horizontal red dashed line depicted in both panels comes from the interpolation of a linear equation obtained with the two rate constants values at the lowest temperatures for the forward isomerization. (Panel a) Isomerization from norcaradiene to cycloheptatriene. The orange stars represent the interpolation and extrapolation of Rubin’s Arrhenius fitting based on the three available experimental data (three black triangles).^12^ The vertical black dashed lines connect the experimental measurements with our CVT/SCT calculations. (Panel b) Isomerization from cycloheptatriene to norcaradiene. The orange stars represent Rubin’s Arrhenius fitting based on previous studies carried out for compound **13**.^13^

Figure 1 shows the unimolecular rate constants for the forward isomerization from norcaradiene to cycloheptatriene (panel a) together with the reverse reaction (panel b)). Solid blue lines represent the rate constants corrected with the multidimensional small curvature tunneling (CVT/SCT), while the dashed lines depict the rate constants without considering tunneling effects (CVT). The three black triangles depicted in panel (a) represent the experimental measurements at 93, 98, and 103 K carried out by Rubin,^12^ which were used to fit to the Arrhenius equation. The latter was used to inter- and extrapolate up to 50 and 500 K, respectively, and is shown as orange stars in panel (a). The vertical black dashed lines connect the experimental measurements to our CVT/SCT calculations with the aim of showing the tunnel regime with which our results are coincidental. Orange stars of the panel (b) represent the Arrhenius fitting for the reverse isomerization proposed by Rubin, and based on data provided by Huisgen for compound **13** (see Supporting Information for further details).^13^ The horizontal red dashed line depicted in both panels of Figure 1 was obtained by interpolating a linear equation derived from the two rate constant values at the lowest temperatures for the forward isomerization. The recrossing coefficient is 1 through the entire range of temperatures, consistent with the absence of variational effects. Thus, the CVT results are identical to those obtained with the transition state theory (TST).^12^

The SCT/CVT calculations depicted in Figure 1 reveal a plateau region from 50 to 80 K (solid blue line), which is the typical behavior in the tunneling-controlled regime. In fact, within this temperature range, there is a significant contribution of heavy-atom tunneling predicted, where the tunneling energy lies on the vibrational ground state of norcaradiene (ν = 0, representative tunneling energies are gathered in Table S6 of the Supporting Information). This behavior can be observed by viewing the red dashed line in panel (a), arising from interpolating the linear equation obtained with two k values at the two lowest temperatures. The slope is practically null and overlaps with the CVT/SCT results up to 80 K, thus showing that up to that temperature the ground state QMT regime dominates. On moving toward increasing T in panel (a), as the red line (ground state QMT) ceases to overlap with the solid blue line (CVT/SCT), and until the latter begins to overlap with the dashed blue line (CVT), the system lies in the shallow tunneling region. During this phase, there still exists a contribution of tunneling effects, but thermally enhanced rate constants start to emerge. This transition phase aligns with the temperature range of 80 < *T* < 190. Interestingly, the only three experiments conducted by Rubin at 93, 98, and 103 K (three black triangles) lie precisely within the shallow tunneling regime. Consequently, all attempts to describe the system by an Arrhenius fitting within this interval becomes impractical (refer to orange stars in panel (a)). Above 190 K, the reaction is completely thermally activated and lies within the expected error of the Arrhenius fitting.

At 50 K, the classical rate constant is 2.4 × 10^–6^ s^–1^, whereas by considering QMT the rate constant rises to 1.8 × 10^3^ s^–1^, approximately 9 orders of magnitude higher. Intuitively, this effect is prominently mirrored by activation free energies^69^ without and with tunneling correction, which are 4.0 and 2.0 kcal mol^–1^, respectively. The free energy of activation is given as Δ*G*^‡^ = −*RT* ln(*k*_B_*T*/*hk*_*i*_), where the *i* denoted for the rate constants could be *C* and *T* for the classical and tunneling corrected, respectively. At this temperature, norcaradiene would be entirely undetectable, having a half-life time of 3.8 × 10^–4^ s. If one ignores tunneling effects, the elusive bicycle would persist longer with a half-life of ∼3.3 days. Within the temperature range 80 < *T* < 190, i.e., in the shallow tunneling regime, the classical and tunneling corrected rate constants range from 2.9 × 10^2^–4.3 × 10^7^ and 2.3 × 10^4^–8.0 × 10^7^ s^–1^, respectively. The experimental results appear in this range, approximately between 90 and 100 K. At 90 K, our CVT/SCT results show a rate constant of 2.3 × 10^4^ s^–1^, whereas the one obtained by Rubin goes down to 9.8 × 10^–5^. Even our classical result is 7 orders of magnitude higher (2.3× 10^2^).

The activation energy derived from the Arrhenius expression obtained by Rubin at 100 K, defined as *E*_a_ = −*R*∂ ln *k*/∂(1/*T*), is 6.1 kcal mol^–1^.^12^ While the *E*_a_ obtained from our CVT/SCT calculation is 2.2 kcal mol^–1^, it is 3.9 kcal mol^–1^ lower. Even without tunneling corrections (CVT), our *E*_a_ lies 2.1 kcal mol^–1^ below that obtained by Rubin. Also, our classical and tunneling corrected activation free energies are notably lower, 4.0 and 3.4 kcal mol^–1^, respectively. Thus, data derived from experiments at 100 K would imply a half-life time of norcaradiene of 3 min. In contrast, our classical and quantum estimations would result in half-lives for norcaradiene of 2.3 × 10^–4^ and 1.7 × 10^–5^ s, respectively, rendering it practically undetectable at such temperatures. Furthermore, the reaction is nonreversible at 100 K since the backward isomerization rate constant is 6.4 × 10^–7^ s^–1^ and the equilibrium constant (*K*_eq_) is 1 × 10^11^, being completely displaced toward cycloheptatriene. As a result and given the accuracy of the present methodology, this assessment convincingly suggests that detection of norcaradiene under the experimental conditions reported would hardly be feasible.

It is worth pointing out the salient discrepancy between such experimental data and the calculations performed in this work. It should be noted that the Arrhenius equation may not accurately describe the system at such low temperatures, especially as the only three experimental measurements agree with the shallow tunneling regime. This conclusion emerges from our classical treatment (CVT) which, even if it deviates notably from experimental data, also exhibits Arrhenius behavior across the entire temperature range. Such a deviation increases significantly when QMT is considered (CVT/SCT), which reveals a non-Arrhenius behavior up to approximately 190 K. This is the expected outcome since it is a well-known fact that QMT leads to deviation from Arrhenius behavior, giving rise to almost temperature-independent rate constants at very low temperatures. In general, it is difficult indeed to compute rate constants agreeing with those obtained experimentally for reactions occurring in a solid matrix, which often leads to lower rate constants than expected. That said, the high discrepancies found in the isomerization of norcaradiene could not be entirely attributed to this effect.

The backward CVT/SCT (solid blue line) calculations displayed in Figure 1 (panel b) were conducted in the same way as for the forward isomerization. Accordingly, tunneling energies are identical for forward and backward reactions as well as the transmission coefficients (κ). The only point that disagrees with those obtained in panel (a) is the barrier height, which in this case is the relative free energy of the transition state with respect to cycloheptatriene. For cycloheptatriene to tunnel backward to its norcaradiene isomer, it should be vibrationally excited to reach the ground-state tunneling energy, which lies on the vibrational ground state of norcaradiene.

The CVT/SCT results for the isomerization from cycloheptatriene to norcaradiene also show a deviation of an Arrhenius behavior, where there is an influence of QMT up to 190 K, as for the forward reaction. That deviation can be inferred from the bifurcation of the CVT/SCT line and the classical CVT results (dashed blue lines) from 190 K. The non-Arrhenius behavior is much less pronounced for the reverse reaction because the tunneling energy lies on the vibrational ground state of norcaradiene which lies 5.0 kcal mol^–1^ above the zero point energy of cycloheptatriene. Thus, for the system to tunnel back to norcaradiene, it should overcome this difference, which becomes realizable only as temperature increases. This fact should therefore be interpreted as meaning that there is no ground-state tunneling at all, namely, from 50 to 190 K is a shallow tunneling region.

The orange stars shown in panel (b) stem from the Arrhenius fitting proposed by Rubin.^12^ This assumption was initially suggested by Huisgen for compound **13**.^13^ Due to the impossibility of detecting norcaradiene, Huisgen associated the ratio between norcaradiene and cycloheptatriene in equilibrium with that detected for compound **13** and assumed that the latter would have the same diene character as norcaradiene. Surprisingly, the Arrhenius fitting resulting from this approach resembles our calculations more than those carried out experimentally [differences between the orange stars and the CVT/SCT results in (panels a,b)].

**Scheme 3:**
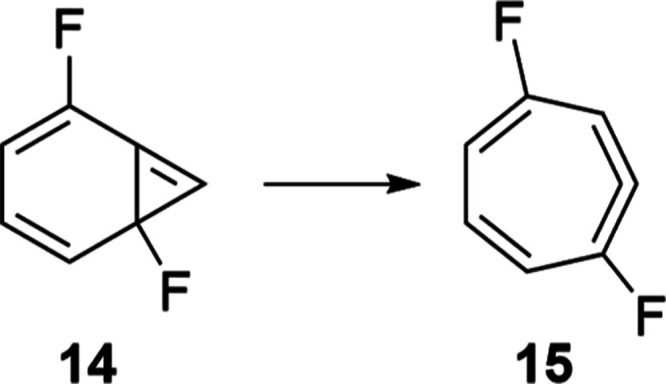
Isomerization of Fluorinated Bycicle **14** to Its Allenic Isomer **15**

Surprised by this unexpected deviation between our theoretical results and those experimentally reported,^12^ further pursuits were taken to validate our calculations, since computing rate constants at such low temperatures for systems of this size is an extremely challenging task. To this end, we have followed the same methodology previously reported to study the isomerization between the fluorinated derivatives bicyclo[4.1.0]hepta-2,4,6-triene (**14**) and the allenic compound cyclohepta-1,2,4,6-tetraene (**15**) (Scheme 3),^45^ which has recently been investigated using state-of-the-art experimental techniques. Accordingly, the same level employed for computing the isomerization between **1** and **2** was applied to its simulation in an argon matrix (SMD) to try to reproduce the experimental conditions.

Our revDSD/CBS energies exhibit discrepancies compared to the calculations conducted at the B3LYP(D3)/def2-TZVP level of theory.^45^ The reaction energy derived with the DFT method including ZPE_Har_ corrections is −15.1 kcal mol^–1^, while our revDSD/CBS calculations in argon (SMD) reveal an energy difference of −10.71 kcal mol^–1^, incorporating ZPE_Anh_ corrections. Although this energy difference diminishes when comparing the barrier height, it remains noticeable, being 3.4 kcal mol^–1^ at the DFT level and 5.4 kcal mol^–1^ with the revDSD method.

The CVT/SCT calculations simulated temperatures down to 5 K, as below this temperature, SCT convergence becomes challenging. From our viewpoint, at temperatures closer to absolute zero, no reliable data can be obtained by using the methodology employed in this work. The comparison of our CVT/SCT calculations with the experimental data conducted by Sander and coworkers^45^ is shown in Table 1. In general, our CVT/SCT rate constants are 1 order of magnitude higher than those obtained experimentally. Assuming that our error in calculating the rate constants at these extremely low temperatures is about 1 order of magnitude, the conclusion regarding the norcaradiene isomerization at 100 K remains unaffected. Yet, if one assumes that the CVT/SCT-based results are 1 order of magnitude faster at 100 K, the rate constant for the isomerization of norcaradiene to cycloheptatriene would be 6.49 × 10^3^ s^–1^, resulting in a half-life of norcaradiene of 1.07 × 10^–4^ s. We can assert that under these conditions norcaradiene would be undetectable.

**Table 1:** Experimental and Theoretical Unimolecular Rate Constants for the forward Isomerization from **14** to **15**

*T* (K)	experimental^45^ (s^–1^)	CVT/SCT (s^–1^)
6	2.0 × 10^–5^	1.4 × 10^–4^
12	2.3 × 10^–5^	3.2 × 10^–4^
25	3.2 × 10^–5^	9.6 × 10^–4^

Aside from this initial recognition of the limitations in our methodology, it should be noted that the maximum error we can incur with our approach occurs when the tunneling energy is equal to that of the least stable isomer’s ground state (ν = 0). Taking into account that norcaradiene leaves the vibrational ground state at temperatures above 90 K, our kinetic results can be deemed reliable for the temperatures at which the experiments were conducted. Hence, for future references regarding the isomerization of norcaradiene to cycloheptatriene in nonpolar environments within the temperature range of 90–500 K, we recommend to resort to the following expression^70^
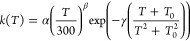
1where α, β, *T*_0_, and γ are fitting parameters, with γ = *E*_a_/*R*, being *R* the ideal gas constant. We obtained a good description of the temperature dependence with fitting parameters α = 3.92 × 10^12^ s^–1^, β = 0.31, γ = 1.60 × 10^3^ K, and *T*_0_ = 80.22 K.

On the other hand, the backward isomerization between norcaradiene and cycloheptatriene is well described by the Arrhenius equation
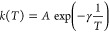
2having obtained a good description of the temperature dependence with parameters A = 1.90 × 10^12^ s^–1^ and γ = 4.28 × 10^3^ K.

## Conclusions

In summary, this theoretical study has provided a comprehensive and updated review of the isomerization between norcaradiene and cycloheptatriene, a subject that has long intrigued chemists in the realm of elusive hydrocarbon species. Noticeable discrepancies with previous experimental data have been identified, especially at low temperatures. The experimentally derived rate constant at 100 K is 3.7 × 10^–3^ s^–1^, while our kinetic calculations, incorporating multidimensional quantum tunneling corrections, yield a value of 6.5 × 10^4^ s^–1^. Even classical TST deviates from the experimental value by 6 orders of magnitude. It is inferred from such results that the exceedingly brief half-life of norcaradiene under these conditions would essentially make it undetectable. These findings not only contribute to the fundamental understanding of this specific isomerization but also provide valuable insights into the influence of heavy-atom tunneling on reactivity, thus opening new avenues for future research in this field.

## Experimental Section

All details about electronic structure and kinetic calculations are gathered in the Supporting Information.

## Data Availability

The data underlying this study are available in the published article and its Supporting Information.

## Supporting Information Available

Electronic structure and kinetic calculations. Geometrical comparison of cycloheptatriene with experimental data. Theoretical and experimental rate constants. Cartesian coordinates of all optimized geometries (PDF)
