# Supplementary material for: Norcaradiene–Cycloheptatriene Equilibrium: A Heavy-Atom Quantum Tunneling Case
Source: J Org Chem. 2024 Jun 18;89(13):9336–43. doi: 10.1021/acs.joc.4c00464 (PMC11232008; doi:10.1021/acs.joc.4c00464)
Supplement: Supplementary file 1 — jo4c00464_si_001.pdf [file jo4c00464_si_001.pdf]

Supporting Information

Norcaradiene-Cycloheptatriene Equilibrium: A Heavy-Atom Quantum Tunneling Case

Juan García de la Concepción,*,a José C. Corchado,*,b Pedro Cintas,[a] and Reyes Babiano[a]

Affiliations:

[a]Juan García de la Concepción*, Pedro Cintas, Reyes Babiano

Departamento de Química Orgánica e Inorgánica, Facultad de Ciencias, and IACYS-Green Chemistry and Sustainable Development Unit, Universidad de Extremadura, 06006 Badajoz, Spain. E-mail: jugarco@unex.es

[b]José C. Corchado*

Departamento de Ingeniería Química y Química Física, Facultad de Ciencias, and ICCAEx, Universidad Extremadura, 06006 Badajoz, Spain. E-mail: corchado@unex.es

Table of Contents:

1.0. Structural Comparison of Theoretical and Experimental Data for Cycloheptatriene S2
2.0. Full Computational Details S2
2.1. Electronic Structure Calculations S3
2.2. Kinetic Calculations S4
*2.3. Note for the Isomerization Between **14** and **15*** S4
3.0. Calculated and Experimental Rate Constants for the Isomerization Between Norcaradiene and Cycloheptatriene S5
3.1. Breakdown of Notes a and b in Table S5 S6
4.0. Calculated and Experimental Rate Constants for the Isomerization Between 14 and 15 S7
5.0. Contribution of Tunneling and Representative Tunneling Energies at Different Temperatures S8
6.0. Absolute Energies and *ZPE* for All Species Studied S9
7.0. Cartesian Coordinates (in Angstroms) for All Optimized Geometries obtained at the revDSD-PBEP86(D3BJ)/jun-cc-pVTZ level of theory. S11
8.0. Cartesian Coordinates Along the Reaction Paths for the Isomerization Reactions S16
*8.1. Isomerization Between **1** and **2**. f (forward) and b (backward) Indicate the Sign of the Reaction Coordinate* S16
*8.2. Isomerization Between **14** and **15**. f (forward) and b (backward) Indicate the Sign of the Reaction Coordinate* S19
9.0. References S21
9.1. Full Citation for Gaussian16 Software Package S22

1.0. Structural Comparison of Theoretical and Experimental Data for Cycloheptatriene

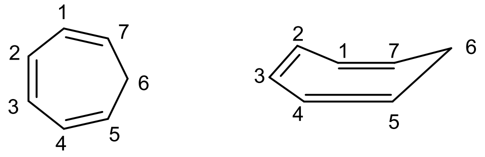

Table S1. Experimental and Calculated Geometrical Parameters of Cycloheptatriene

Parameter	revDSD-PBEP86/jun-cc-pVTZ	Experimental
r (C6-C7)	1.503	1.505
r (C1-C2)	1.447	1.446
r (C1-C7)	1.349	1.356
A (C5-C6-C7)	107.7	105.0
A (C6-C7-C1)	121.3	121.8
A (C1-C2-C3)	124.8	127.2
A (C2-C3-C4)	125.5	119.8

2.0. Full Computational Details

2.1. Electronic Structure Calculations

Norcaradiene, cycloheptatriene and the saddle point linking the two isomers were optimized with the double hybrid revDSD-PBEP86[1,2] in combination with the D3BJ empirical dispersion correction[3,4] and the correlation consistent basis set jun-cc-pVTZ[5,6,7] in vacuum. Hessian evaluation was carried out at the same level of theory finding none and one imaginary frequencies for energy minima and saddle point, respectively. Energy refinements were carried out by extrapolating to the complete basis set limit with a mixed Gaussian/exponential expression for cardinal numbers *n* = 3, 4 and 5 solving the following system of equations:[8,9,10]

$$\begin{cases} E\ (n_1) = E_{CBS} + Ae^{-(n_1-1)} + Be^{-(n_1-1)^2} \\ E\ (n_2) = E_{CBS} + Ae^{-(n_2-1)} + Be^{-(n_2-1)^2} \\ E\ (n_3) = E_{CBS} + Ae^{-(n_3-1)} + Be^{-(n_3-1)^2} \end{cases}$$

To validate the level of theory, we also corrected the electronic energies with the CCSD(T)-F12[11.12] on the revDSD-PBEP86(D3BJ)/jun-cc-pVTZ geometries (abbreviated as revDSD). As auxiliary and special orbital basis set, the cc-pVTZ-F12-CABS and cc-pVTZ-F12 were used. Comparative results are gathered in Table S2.

Table S2. Relative Electronic Energies of the Stationary Points with Respect to the Less Stable Isomer in kcal/mol and *T_1* Diagnostic Taken from the Coupled Cluster Calculations

Structure	revDSD-PBEP86/ jun-cc-pVTZ	revDSD-PBEP86/ CBS	CCSD(T)-F12/ cc-pVTZ-F12	T_1 diagnostic
Norcaradiene	0.00	0.00	0.00	0.01
Saddle point	4.62	4.83	4.98	0.01
Cycloheptatriene	-5.61	-5.34	-5.73	0.01

As inferred from Table S2 the revDSD-PBEP86(D3BJ)/CBS energies give very similar results to those calculated with Coupled Cluster. The result exhibiting the largest deviation from the CCSD(T)-F12 energies is the relative stability between the isomers (0.39 kcal/mol). This small difference is not critical for the conclusions attained in this work that focuses mainly on the kinetics of the isomerization from norcaradiene to cyclocheptatriene. The T_1 diagnostic reveals that static correlation calculations are unnecessary for this system. Accordingly, our reference level of theory through this work is revDSD-PBEP86(D3BJ)/CBS for the calculation of energies on revDSD-PBEP86(D3BJ)/jun-cc-pVTZ geometries.

Since high-frequency vibrational modes in the transition state are important for predicting quantitative rate constants at low temperatures, anharmonic zero-point vibrational energies corrections (ZPE_{Anh}) were evaluated at revDSD-PBEP86(D3BJ)/jun-cc-pVTZ within vibrational perturbative theory to second order (VPT2).[13] For calculating the scaling factor to account for high frequency anharmonicity, the following expression was employed:[14]

$$\omega_{Anh} = \lambda^{ZPE}\omega_{Har}$$

where ω_{Anh} and ω_{Har} are the anharmonic and harmonic ZPE energies computed at the above-mentioned level of theory, respectively. λ^{ZPE} is the scaling factor used to correct harmonicities. The results obtained are shown in Table S3.

Table S3. Harmonic and Anharmonic Zero Point Vibrational Energies in kcal/mol and $\omega_{Anh}/\omega_{Har}$ (Freq. scal.)

Structure	ω_{Har}	ω_{Anh}	Freq scal
Norcaradiene (**1**)	80.424	79.442	0.988
Saddle point (**TS**)	80.633	79.703	0.988
Cycloheptatriene (**2**)	79.814	78.875	0.988

2.2. Kinetic Calculations

In order to reproduce the experimental results conducted by Rubin,[15] our kinetic calculations were performed taking solvent effects into consideration. Thus, all calculations conducted up to this point have been repeated using the continuum solvation model (SMD)[16,17] in cyclohexane.

For kinetic calculations, we used the canonical variational transition state theory (CVT)[18] employing the small curvature tunneling approximation (SCT)[18] across the temperature (T) range of 50–500 K, and using the Pilgrim software.[19] The expression for unimolecular rate constants is provided by:

$$k^{CVT/SCT}(T) = \kappa^{SCT}\Gamma^{CVT}\frac{K_B T}{h}\frac{Q^{VT}}{Q^R}e^{(\frac{-E^{VT}}{K_B T})}$$

where *R* represents the ideal gas constant, κ^{SCT} is the small curvature multidimensional tunneling transmission coefficient, and Γ^{CVT} is the canonical variational transition state recrossing coefficient. This coefficient is defined as k^{CVT}/k^{TST}, where k^{TST} is the rate coefficient of conventional transition state theory, and k^{CVT} is the rate coefficient of canonical variational transition state theory. Additionally, K_B stands for the Boltzmann constant, and *h* denotes the Planck constant.

Q^{VT} and Q^{R} are the total partition functions of the variational transition state and the reactant, respectively, while E^{VT} represents the potential energy of the variational transition state.

The energies along the minimum energy path (MEP), obtained using the revDSD-PBEP86(D3BJ)/jun-cc-pVTZ method in cyclohexane (SMD), were corrected with the revDSD-PBEP86(D3BJ)/CBS in cyclohexane (SMD), employing the interpolated single-point energies (ISPE) algorithm. For correcting the MEP we used the two isomers, the saddle point and three points in the backward direction (S<0), as well as three additional points in the forward direction (S>0).

To account for the high frequency anharmonicities, we multiplied the harmonic frequencies of the norcaradiene and cycloheptatriene by the scaling factor 0.988. The same scaling factor was used for the saddle point and the whole MEP.

*2.3. Note for the Isomerization Between **14** and **15***

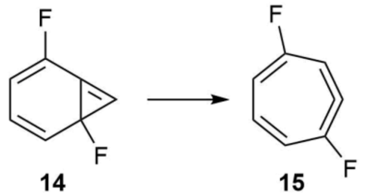

Figure S1. Isomerization of fluorinated bycicle **14** to allene **15**.

All the electronic and kinetic calculations for the isomerization between **14** and **15** have been carried out with the same methodology as detailed in sections 2.1 and 2.2, but changing the solvent to argon (SMD).

3.0. Calculated and Experimental Rate Constants for the Isomerization Between Norcaradiene and Cycloheptatriene

Table S4. Calculated and Experimental Rate Constants in s^{-1} for the Isomerization Between Norcaradiene and Cycloheptatriene, Calculated Free Energy of Reaction in kcal/mol and Equilibrium Constants

	This work						Experimental	
	Towards cycloheptatriene		Towards norcaradiene		ΔG_{reacc}	K_{eq}	Towards cycloheptatriene[a]	Towards norcaradiene[b]
T (K)	CVT	CVT/SCT	CVT	CVT/SCT				
50	2.4E-06	1.8E+03	2.6E-28	2.0E-19	-5.0	9.1E+21	2.3E-17	2.1E-40
60	2.5E-03	3.0E+03	1.2E-21	1.5E-15	-5.0	2.0E+18	1.3E-12	1.2E-31
70	3.7E-01	5.1E+03	7.3E-17	1.0E-12	-5.0	5.0E+15	3.0E-09	2.0E-25
80	1.6E+01	9.8E+03	2.8E-13	1.8E-10	-5.0	5.5E+13	1.0E-06	9.8E-21
90	2.9E+02	2.3E+04	1.8E-10	1.4E-08	-5.0	1.7E+12	9.8E-05	4.3E-17
100	3.1E+03	6.5E+04	3.0E-08	6.4E-07	-5.0	1.0E+11	3.7E-03	3.5E-14
120	1.1E+05	6.0E+05	7.0E-05	3.9E-04	-5.0	1.5E+09	8.7E-01	8.3E-10
140	1.4E+06	4.3E+06	1.8E-02	5.6E-02	-5.1	7.7E+07	4.3E+01	1.1E-06
160	9.5E+06	2.2E+07	1.1E+00	2.6E+00	-5.1	8.3E+06	7.9E+02	2.4E-04
180	4.3E+07	8.0E+07	2.9E+01	5.5E+01	-5.1	1.5E+06	7.7E+03	1.6E-02
200	1.4E+08	2.4E+08	3.9E+02	6.4E+02	-5.1	3.7E+05	4.7E+04	4.6E-01
220	3.9E+08	5.9E+08	3.3E+03	4.9E+03	-5.1	1.2E+05	2.1E+05	7.2E+00
240	9.1E+08	1.3E+09	1.9E+04	2.7E+04	-5.1	4.7E+04	7.2E+05	7.1E+01
260	1.8E+09	2.4E+09	8.6E+04	1.1E+05	-5.2	2.1E+04	2.1E+06	4.9E+02
280	3.4E+09	4.3E+09	3.1E+05	4.0E+05	-5.2	1.1E+04	5.1E+06	2.6E+03
298.15	5.4E+09	6.9E+09	8.0E+05	1.5E+06	-5.2	4.7E+03	1.0E+07	9.6E+03
300	5.8E+09	7.1E+09	9.4E+05	1.E+06	-5.2	6.1E+03	1.1E+07	1.1E+04
320	9.2E+09	1.1E+10	2.5E+06	3.0E+06	-5.2	3.7E+03	2.2E+07	3.8E+04
340	1.4E+10	1.E+10	5.9E+06	7.0E+06	-5.2	2.4E+03	4.0E+07	1.2E+05
360	2.0E+10	2.34E+10	1.3E+07	1.5E+07	-5.3	1.6E+03	6.8E+07	3.1E+05
380	2.8E+10	3.2E+10	2.5E+07	2.9E+07	-5.3	1.1E+03	1.1E+08	7.5E+05
400	3.8E+10	4.3E+10	4.7E+07	5.3E+07	-5.3	8.1E+02	1.7E+08	1.7E+06
420	5.0E+10	5.5E+10	8.2E+07	9.1E+07	-5.4	6.1E+02	2.5E+08	3.4E+06
460	8.0E+10	8.7E+10	2.2E+08	2.4E+08	-5.4	3.7E+02	4.9E+08	1.2E+07
480	9.8E+10	1.1E+11	3.3E+08	3.6E+08	-5.4	3.0E+02	6.6E+08	2.1E+07
500	1.2E+11	1.3E+11	4.9E+08	5.3E+08	-5.5	2.4E+02	8.6E+08	3.4E+07

[a]Inter- and extrapolation of the Rubin's Arrhenius Fitting obtained with his three measurements at 93, 98, and 103 K.[15]

[b]Arrhenius fitting for the reverse isomerization proposed by Rubin, and based on data reported by Huisgen for compound **13**.[15,20]

3.1. Breakdown of Notes a and b in Table S5.

The assumption reached by Huisgen to estimate the population between norcaradiene and cycloheptatriene is based on bicyclo[4.2.0]octa-2,4-diene, as norcaradiene could not be detected. He stated the following verbatim:

The Diels-Alder adducts of cycloheptatriene (C) are structurally derived from norcaradiene (D). Kinetic measurements on the reaction with tetracyanoethylene gave k_d values that were proportional to the dienophile concentration over the entire range measured. This means that the establishment of the equilibrium (C)+(D) is fast in comparison with the Diels-Alder reaction. The intermediate cannot be detected kinetically in this case. If it is assumed that the diene activity of (D) can be equated to that of (B) (equal k_2 values for the addition of tetracyanoethylene to the cyclohexadiene portion of the two molecules), a 0.1 % equilibrium concentration of (D) at 20 "C would lead to kd values of the order found.

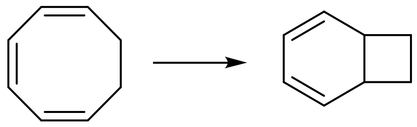

(A) (B)

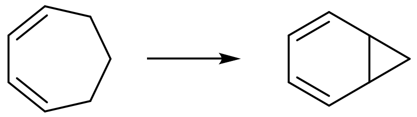

(C) (D)

Then, based on the above-mentioned assumption, Rubin stated:

It has been suggested (quoting Huisgen) that the equilibrium concentration of 1 (referred to norcaradiene) *in 2* (referred to cycloheptatriene) *at 20 °C is 0.1%. Using the equilibrium constant based on this proposal, the free energy difference between 1 and 2 at 25 °C is of the order of -4 kcal/mol and $\Delta G^{\ddagger}_{1\rightarrow 2}$ ≈11 kcal/mol. Assuming that the entropy of activation for 2 → 1 is close to zero, the approximate Arrhenius expression becomes*

$$k_{2\rightarrow 1} \approx 6x10^{12}e^{-12000/RT}$$

4.0. Calculated and Experimental Rate Constants for the Isomerization Between 14 and 15

Table S5. Calculated and Experimental Rate Constants in s^{-1} for the Isomerization Between 14 and 15, Calculated Free Energy of Reaction in kcal/mol and Equilibrium Constants

	This work						Merini et al. 2023	
	Towards **15**		Towards **14**		ΔG_{reacc}	K_{eq}	Towards **15**	Towards **14**
T (K)	CVT	CVT/SCT	CVT	CVT/SCT				
5	4.7E-225	1.1E-04	2.4E-693	5.7E-473	-10.7	---	---	---
6	9.5E-186	1.4E-04	5.3E-576	7.7E-395	-10.7	---	2.0E-5	---
10	4.5E-107	2.5E-04	3.0E-341	1.7E-238	-10.7	1.5E+234	---	---
12	2.2E-87	3.2E-04	1.6E-282	2.2E-199	-10.7	1.4E+195	2.3E-5	---
20	6.2E-48	6.6E-04	4.9E-165	5.1E-121	-10.7	1.3E+117	---	---
25	4.6E-36	9.6E-04	9.2E-130	1.9E-97	-10.7	5.0E+93	3.7E-03	---
30	3.8E-28	1.4E-03	3.1E-106	1.1E-81	-10.7	1.2E+78	---	---
40	3.3E-18	2.8E-03	8.7E-77	7.4E-62	-10.7	3.8E+58	---	---
50	3.1E-12	6.2E-03	4.2E-59	8.3E-50	-10.7	7.5E+46	---	---
60	3.1E-08	1.6E-02	2.6E-47	1.4E-41	-10.7	1.2E+39	---	---
70	2.3E-05	5.7E-02	7.2E-39	1.8E-35	-10.7	3.1E+33	---	---
80	3.2E-03	3.2E-01	1.6E-32	1.5E-30	-10.7	2.1E+29	---	---
90	1.6E-01	2.7E+00	1.3E-27	2.3E-26	-10.7	1.2E+26	---	---
100	3.5E+00	2.5E+01	1.2E-23	8.7E-23	-10.7	2.9E+23	---	---
120	3.7E+02	1.2E+03	1.0E-17	3.3E-17	-10.7	3.7E+19	---	---
140	1.1E+04	2.4E+04	1.7E-13	3.9E-13	-10.8	6.1E+16	---	---
160	1.3E+05	2.4E+05	2.7E-10	4.8E-10	-10.8	5.0E+14	---	---
180	9.6E+05	1.5E+06	8.0E-08	1.3E-07	-10.8	1.2E+13	---	---
200	4.7E+06	6.8E+06	7.8E-06	1.1E-05	-10.8	6.1E+11	---	---
220	1.7E+07	2.4E+07	3.3E-04	4.4E-04	-10.8	5.3E+10	---	---
240	5.2E+07	6.7E+07	7.5E-03	9.6E-03	-10.8	7.0E+09	---	---
260	1.3E+08	1.6E+08	1.1E-01	1.3E-01	-10.8	1.2E+09	---	---
280	3.0E+08	3.5E+08	1.0E+00	1.2E+00	-10.8	2.9E+08	---	---
298.15	5.6E+08	6.6E+08	6.3E+00	7.3E+00	-10.8	8.9E+07	---	---
300	6.0E+08	7.0E+08	7.4E+00	8.7E+00	-10.8	8.0E+07	---	---
320	1.1E+09	1.3E+09	4.2E+01	4.8E+01	-10.9	2.6E+07	---	---
340	1.9E+09	2.1E+09	1.9E+02	2.2E+02	-10.9	9.8E+06	---	---
360	3.1E+09	3.4E+09	7.5E+02	8.4E+02	-10.9	4.1E+06	---	---
380	4.8E+09	5.2E+09	2.5E+03	2.8E+03	-10.9	1.9E+06	---	---
400	7.0E+09	7.7E+09	7.6E+03	8.3E+03	-10.9	9.2E+05	---	---
420	1.0E+10	1.1E+10	2.1E+04	2.2E+04	-10.9	4.9E+05	---	---
440	1.4E+10	1.5E+10	5.1E+04	5.5E+04	-10.9	2.7E+05	---	---
460	1.9E+10	2.0E+10	1.2E+05	1.2E+05	-11.0	1.6E+05	---	---
480	2.5E+10	2.6E+10	2.5E+05	2.7E+05	-11.0	9.8E+04	---	---
500	3.2E+10	3.3E+10	5.0E+05	5.3E+05	-11.0	6.3E+04	---	---

5.0. Contribution of Tunneling and Representative Tunneling Energies at Different Temperatures.

Table S6. Representative Tunneling Energies (RTE in kcal/mol), Contribution of Tunneling (I1%), and Contribution of Non-classical Reflection

T (K)	%I1	%I2	RTE
50	100	0	79.618[a]
60	100	0	79.618[a]
70	100	0	79.618[a]
80	99.9	0.1	79.618[a]
90	99.15	0.85	80.275
100	96.75	3.25	82.136
120	87.4	12.6	83.264
140	76.74	23.26	83.418
160	67.27	32.73	83.506
180	59.34	40.66	83.564
200	52.78	47.22	83.606
220	47.34	52.66	83.643
240	42.79	57.21	83.684
260	38.96	61.04	83.725
280	35.71	64.29	83.758
300	32.91	67.09	83.784
320	30.49	69.51	83.806
340	28.38	71.62	83.825
360	26.53	73.47	83.844
380	24.89	75.11	83.86
400	23.43	76.57	83.876
420	22.12	77.88	83.889
460	19.89	80.11	83.915
480	18.93	81.07	83.927
500	18.05	81.95	83.938

[a] RTE values from 50 to 80 agree with the ZPE of norcaradiene.

6.0. Absolute Energies and *ZPE* for all Species Studied.

Table S7. CCSD(T)-F12/cc-pVTZ-F12 energies (in Hartrees) on revDSD geometries in vacuum

Structure	*E*
**1**	-271.089676
**TS**	-271.08174
**2**	-271.098809

Table S8. revDSD energies in vacuum (in Hartrees) on jun-cc-pVTZ geometries and *ZPE* energies in kcal/mol.

Structure	jun-cc-pV*n*Z	*E*	ZPE_{Har}	*ZPEAnh*
**1**	3	-271.055819	80.424	79.442
	4	-271.109077	---	---
	5	-271.127031	---	---
**TS**	3	-271.048451	80.633	79.703
	4	-271.101499	---	---
	5	-271.11938	---	---
**2**	3	-271.064765	79.814	78.875
	4	-271.117767	---	---
	5	-271.135609	---	---

Table S9. revDSD energies in cyclohexane (SMD) for the stationary points and the six points along the minimum energy path used for the correction of the energy profile for the isomerization between 1 and 2. f (forward) and b (backward) indicate the sign of the reaction coordinate. *ZPE* are given in kcal/mol.

Structure	jun-cc-pV*n*Z	*E*	ZPE_{Har}
**1**	3	-271.064535	80.584
	4	-271.117814	---
	5	-271.135775	---
**b180**	3	-271.063801	---
	4	-271.117064	---
	5	-271.135026	---
**b120**	3	-271.061375	---
	4	-271.114577	---
	5	-271.132516	---
**b60**	3	-271.058464	---
	4	-271.111596	---
	5	-271.129507	---
**TS**	3	-271.057191	79.795
	4	-271.110257	---
	5	-271.128144	---
**f47**	3	-271.058048	---
	4	-271.111077	---
	5	-271.128953	---
**f93**	3	-271.060363	---
	4	-271.113376	---
	5	-271.131246	---
**f140**	3	-271.0637	---
	4	-271.116715	---
	5	-271.134581	---
**2**	3	-271.07266	80.392
	4	-271.12568	---
	5	-271.143529	---

Table S10. revDSD energies in cyclohexane (SMD) for the stationary points and the six points along the minimum energy path used for the correction of the energy profile for the isomerization between 14 and 15. f (forward) and b (backward) indicate the sign of the reaction coordinate. *ZPE* are given in kcal/mol.

Structure	jun-cc-pV*n*Z	*E*	ZPE_{Har}
**14**	3	-468.089487	54.886
	4	-468.1787	---
	5	-468.210011	---
**b360**	3	-468.089216	---
	4	-468.178436	---
	5	-468.209747	---
**b240**	3	-468.08752	---
	4	-468.176734	---
	5	-468.208042	---
**b120**	3	-468.083202	---
	4	-468.172348	---
	5	-468.203631	---
**$TS_{14\rightarrow15}$**	3	-468.079418	
	4	-468.168461	54.027
	5	-468.199703	---
**f77**	3	-468.081028	---
	4	-468.170004	---
	5	-468.201225	---
**f153**	3	-468.084764	---
	4	-468.173704	---
	5	-468.204915	---
**f230**	3	-468.089271	---
	4	-468.178194	---
	5	-468.209404	---
**15**	3	-468.107621	55.355
	4	-468.196485	---
	5	-468.227703	---

7.0. Cartesian Coordinates (in Angstroms) for All Optimized Geometries obtained at the revDSD-PBEP86(D3BJ)/jun-cc-pVTZ level of theory.

Norcaradiene in vacuum

C	1.44501000	0.20300000	0.72699700
C	1.44501000	0.20300000	-0.72699700
C	0.35454000	-0.17262500	-1.42689400
C	0.35454000	-0.17262500	1.42689400
C	-0.94418300	-0.40147900	-0.77943000
C	-0.94418300	-0.40147900	0.77943000
C	-1.59404300	0.70795000	0.00000000
H	2.37674700	0.39803500	1.24494900
H	2.37674700	0.39803500	-1.24494900
H	0.42222900	-0.33205000	-2.49758700
H	0.42222900	-0.33205000	2.49758700
H	-1.61811300	-1.09263000	-1.27120400
H	-1.61811300	-1.09263000	1.27120400
H	-1.09212300	1.66712500	0.00000000
H	-2.67515700	0.73836800	0.00000000

Zero-point correction= 0.128497 (Hartree/Particle)
Thermal correction to Energy= 0.133963
Thermal correction to Enthalpy= 0.134908
Thermal correction to Gibbs Free Energy= 0.099689
Sum of electronic and zero-point Energies= -270.927321
Sum of electronic and thermal Energies= -270.921855
Sum of electronic and thermal Enthalpies= -270.920911
Sum of electronic and thermal Free Energies= -270.956129

Cycloheptatriene in vacuum

C	-1.41379300	0.16592400	-0.68079700
C	-1.41379300	0.16592400	0.68079700
C	-0.32735400	-0.28846200	1.52238900
C	-0.32735400	-0.28846200	-1.52238900
C	0.98117000	-0.18077400	1.21381300
C	0.98117000	-0.18077400	-1.21381300
C	1.43424500	0.58178700	0.00000000
H	-2.34943100	0.40542400	-1.17745000
H	-2.34943100	0.40542400	1.17745000
H	-0.60408900	-0.76293300	2.45926800
H	-0.60408900	-0.76293300	-2.45926800
H	1.72427000	-0.64010100	1.85764900
H	1.72427000	-0.64010100	-1.85764900
H	0.96336200	1.57216000	0.00000000
H	2.51557900	0.71615200	0.00000000

Zero-point correction= 0.128163 (Hartree/Particle)
Thermal correction to Energy= 0.133899
Thermal correction to Enthalpy= 0.134843

Thermal correction to Gibbs Free Energy= 0.099070
Sum of electronic and zero-point Energies= -270.936602
Sum of electronic and thermal Energies= -270.930866
Sum of electronic and thermal Enthalpies= -270.929922
Sum of electronic and thermal Free Energies= -270.965695

Saddle point in vacuum

C	1.41592200	0.20804800	0.70810200
C	1.41592200	0.20804900	-0.70810200
C	0.34346700	-0.25302900	-1.44519200
C	0.34346700	-0.25302900	1.44519200
C	-0.97383600	-0.31267600	-0.94229400
C	-0.97383600	-0.31267600	0.94229400
C	-1.48090700	0.71114000	0.00000000
H	2.36814600	0.34692000	1.20827800
H	2.36814600	0.34692000	-1.20827900
H	0.52536500	-0.66163500	-2.43398000
H	0.52536500	-0.66163500	2.43398000
H	-1.69763400	-0.96876600	-1.41191300
H	-1.69763400	-0.96876600	1.41191300
H	-0.92596300	1.64600100	0.00000000
H	-2.55740000	0.84222500	0.00000000

Zero-point correction= 0.127191 (Hartree/Particle)
Thermal correction to Energy= 0.132379
Thermal correction to Enthalpy= 0.133324
Thermal correction to Gibbs Free Energy= 0.098579
Sum of electronic and zero-point Energies= -270.921260
Sum of electronic and thermal Energies= -270.916072
Sum of electronic and thermal Enthalpies= -270.915128
Sum of electronic and thermal Free Energies= -270.949873

Norcaradiene in cyclohexane (smd)

C	1.44519100	0.20441900	0.72686100
C	1.44519100	0.20441900	-0.72686100
C	0.35429300	-0.17252500	-1.42655000
C	0.35429300	-0.17252500	1.42655000
C	-0.94476300	-0.40085400	-0.78036000
C	-0.94476300	-0.40085400	0.78036000
C	-1.59346700	0.70771100	0.00000000
H	2.37755300	0.39842600	1.24465000
H	2.37755300	0.39842600	-1.24465000
H	0.42290800	-0.33523700	-2.49698400
H	0.42290800	-0.33523700	2.49698400
H	-1.61903700	-1.09139500	-1.27195000
H	-1.61903700	-1.09139500	1.27195000
H	-1.09286000	1.66790700	0.00000000
H	-2.67482600	0.73666200	0.00000000

Zero-point correction= 0.128418 (Hartree/Particle)
Thermal correction to Energy= 0.133889
Thermal correction to Enthalpy= 0.134833
Thermal correction to Gibbs Free Energy= 0.099609
Sum of electronic and zero-point Energies= -270.936117
Sum of electronic and thermal Energies= -270.930646
Sum of electronic and thermal Enthalpies= -270.929702
Sum of electronic and thermal Free Energies= -270.964926

Cycloheptatriene in cyclohexane (smd)

C	-1.41525200	0.16528400	-0.68095700
C	-1.41525200	0.16528400	0.68095700
C	-0.32795300	-0.28743000	1.52222900
C	-0.32795300	-0.28743000	-1.52222900
C	0.98099600	-0.17982400	1.21336300
C	0.98099600	-0.17982400	-1.21336300
C	1.43600800	0.58160400	0.00000000
H	-2.35150600	0.40209600	-1.17809300
H	-2.35150600	0.40209600	1.17809300
H	-0.60466300	-0.76110000	2.45980500
H	-0.60466300	-0.76110000	-2.45980500
H	1.72410300	-0.63836600	1.85813200
H	1.72410300	-0.63836600	-1.85813200
H	0.96946600	1.57415200	0.00000000
H	2.51780600	0.71117800	0.00000000

Zero-point correction= 0.128113 (Hartree/Particle)
Thermal correction to Energy= 0.133854
Thermal correction to Enthalpy= 0.134799
Thermal correction to Gibbs Free Energy= 0.099015
Sum of electronic and zero-point Energies= -270.944547
Sum of electronic and thermal Energies= -270.938805
Sum of electronic and thermal Enthalpies= -270.937861
Sum of electronic and thermal Free Energies= -270.973645

Saddle point in cyclohezane (smd)

C	1.41623900	0.20961900	0.70758800
C	1.41623900	0.20961900	-0.70758800
C	0.34276300	-0.25312600	-1.44530800
C	0.34276300	-0.25312600	1.44530800
C	-0.97446100	-0.30972600	-0.94609000
C	-0.97446100	-0.30972600	0.94608900
C	-1.48046200	0.70991600	0.00000000
H	2.36889900	0.34793500	1.20775500
H	2.36889900	0.34793500	-1.20775500
H	0.52701000	-0.66784600	-2.43146400
H	0.52701100	-0.66784600	2.43146400

H	-1.69825700	-0.96600000	-1.41552200
H	-1.69825700	-0.96600000	1.41552200
H	-0.92811900	1.64658500	0.00000000
H	-2.55721700	0.83888000	0.00000000

Zero-point correction= 0.127162 (Hartree/Particle)
Thermal correction to Energy= 0.132351
Thermal correction to Enthalpy= 0.133295
Thermal correction to Gibbs Free Energy= 0.098548
Sum of electronic and zero-point Energies= -270.930029
Sum of electronic and thermal Energies= -270.924840
Sum of electronic and thermal Enthalpies= -270.923896
Sum of electronic and thermal Free Energies= -270.958644

Compound 14 in argon (smd)

C	1.18606400	1.20444800	0.24342200
C	-0.02443100	1.81819300	0.17845800
C	-1.27909000	1.13093300	-0.07173100
C	-0.12159600	-0.88774500	0.22207600
C	1.22136400	-0.25194400	0.05965200
C	-1.33890300	-0.22112700	-0.08083700
H	-0.07464600	2.89598800	0.28335400
H	1.09956300	-1.77945500	1.94742800
H	-2.18751400	1.70616300	-0.20154000
F	2.04141700	-0.62947600	-1.00855400
F	-2.47199600	-0.89766600	-0.30842700
H	2.10103100	1.77894500	0.32813200
C	0.84605600	-1.26898600	1.03153600

Zero-point correction= 0.087467 (Hartree/Particle)
Thermal correction to Energy= 0.094369
Thermal correction to Enthalpy= 0.095313
Thermal correction to Gibbs Free Energy= 0.056132
Sum of electronic and zero-point Energies= -468.002020
Sum of electronic and thermal Energies= -467.995118
Sum of electronic and thermal Enthalpies= -467.994174
Sum of electronic and thermal Free Energies= -468.033355

Compound 15 in argon (smd)

C	1.04477800	1.13595300	-0.21211600
C	-0.21840100	1.68381800	0.19120300
C	-1.41586600	1.04737300	0.36639800
C	-0.54491400	-1.17946400	0.41479400
C	1.49601000	-0.14288200	-0.05352300
C	-1.50954600	-0.34867600	0.04732300
H	-0.21754100	2.76097000	0.33025500
H	1.29936300	-1.86493700	1.28335100
H	-2.29435100	1.61197100	0.66227300

F	2.76438000	-0.40734600	-0.42545300
F	-2.57936500	-0.74848300	-0.67444100
H	1.77443400	1.84013100	-0.59813300
C	0.77676600	-1.18706900	0.61613900

Zero-point correction= 0.088214 (Hartree/Particle)
Thermal correction to Energy= 0.095218
Thermal correction to Enthalpy= 0.096162
Thermal correction to Gibbs Free Energy= 0.056672
Sum of electronic and zero-point Energies= -468.019407
Sum of electronic and thermal Energies= -468.012403
Sum of electronic and thermal Enthalpies= -468.011458
Sum of electronic and thermal Free Energies= -468.050949

Saddle point linking compounds 14 and 15 in argon (smd)

C	1.11065400	1.22315900	0.02687800
C	-0.14203500	1.78914900	0.17803700
C	-1.36213700	1.08198000	0.07626200
C	-0.25099000	-0.96301100	0.40662200
C	1.34176100	-0.17614700	0.04202400
C	-1.37444800	-0.29227600	-0.01078300
H	-0.20175000	2.87041700	0.22637600
H	1.25088100	-1.51487300	1.97150600
H	-2.29337200	1.62876200	-0.01611100
F	2.22822300	-0.63890200	-0.88982400
F	-2.44114100	-0.95148000	-0.50530000
H	1.94446100	1.86111800	-0.24943800
C	0.87987000	-1.08485100	1.05159100

Zero-point correction= 0.086098 (Hartree/Particle)
Thermal correction to Energy= 0.092764
Thermal correction to Enthalpy= 0.093709
Thermal correction to Gibbs Free Energy= 0.054985
Sum of electronic and zero-point Energies= -467.993321
Sum of electronic and thermal Energies= -467.986654
Sum of electronic and thermal Enthalpies= -467.985710
Sum of electronic and thermal Free Energies= -468.024434

8.0. Cartesian Coordinates Along the Reaction Paths for the Isomerization Reactions

*8.1. Isomerization between **1** and **2**. f (forward) and b (backward) indicate the sign of the reaction coordinate.*

b 60 in clyclohexane (smd)

C	1.41892000	0.20412300	0.71401200
C	1.41892000	0.20412300	-0.71401200
C	0.34670100	-0.24489000	-1.43908000
C	0.34670100	-0.24489000	1.43908000
C	-0.97170100	-0.32698500	-0.89388200
C	-0.97170100	-0.32698500	0.89388200
C	-1.49171400	0.73402200	0.00000000
H	2.37104300	0.34110000	1.21495700
H	2.37104300	0.34110000	-1.21495700
H	0.50281200	-0.61220800	-2.44834200
H	0.50281200	-0.61220800	2.44834200
H	-1.69610500	-0.98103400	-1.36434400
H	-1.69610500	-0.98103400	1.36434400
H	-0.93199600	1.66444800	0.00000000
H	-2.56808000	0.85747800	0.00000000

b 120 in clyclohexane (smd)

C	1.42299600	0.19653500	0.71983700
C	1.42299600	0.19653500	-0.71983700
C	0.34830300	-0.23590900	-1.43291400
C	0.34830300	-0.23590900	1.43291400
C	-0.96816500	-0.34509300	-0.84246000
C	-0.96816500	-0.34509300	0.84246000
C	-1.50464500	0.75643000	0.00000000
H	2.37246300	0.33888900	1.22376400
H	2.37246300	0.33888900	-1.22376400
H	0.47425900	-0.54882700	-2.46418200
H	0.47425900	-0.54882700	2.46418200
H	-1.69251900	-0.99368600	-1.31977600
H	-1.69251900	-0.99368600	1.31977600
H	-0.93783100	1.68089900	0.00000000
H	-2.58064900	0.87513600	0.00000000

b 180 in clyclohexane (smd)

C	1.43115100	0.18317900	0.72478500
C	1.43115100	0.18317900	-0.72478500
C	0.34739700	-0.22128600	-1.42687800
C	0.34739700	-0.22128600	1.42687800
C	-0.96354100	-0.36266100	-0.79733200
C	-0.96354100	-0.36266100	0.79733200
C	-1.52155900	0.77472900	0.00000000
H	2.37444400	0.33986300	1.23553800
H	2.37444400	0.33986300	-1.23553800
H	0.43798600	-0.46280300	-2.48054600
H	0.43798600	-0.46280300	2.48054600
H	-1.68443300	-1.00863600	-1.28291500
H	-1.68443300	-1.00863600	1.28291500
H	-0.94998900	1.69500100	0.00000000
H	-2.59753500	0.88727700	0.00000000

f 47 in clyclohexane (smd)

C	1.41569100	0.21391400	0.70227500
C	1.41569100	0.21391400	-0.70227700
C	0.33944900	-0.25867900	-1.45061500
C	0.33944900	-0.25867900	1.45061300
C	-0.97554100	-0.29528100	-0.98664100
C	-0.97554100	-0.29528100	0.98664100
C	-1.47131800	0.69159200	0.00000000
H	2.36758100	0.35753400	1.20289700
H	2.36758100	0.35753300	-1.20289900
H	0.54488800	-0.70687700	-2.41796600
H	0.54488900	-0.70687600	2.41796500
H	-1.69916800	-0.94888900	-1.46103400
H	-1.69916800	-0.94888900	1.46103400
H	-0.92471000	1.63316200	0.00000000
H	-2.54827400	0.82611700	0.00000000

f 93 in clyclohexane (smd)

C	1.41514600	0.21727900	0.69711200
C	1.41514600	0.21727800	-0.69711500
C	0.33579500	-0.26483900	-1.45649700
C	0.33579600	-0.26483800	1.45649600
C	-0.97683500	-0.28167100	-1.02568600
C	-0.97683500	-0.28167200	1.02568500
C	-1.46322600	0.67242900	0.00000000
H	2.36522100	0.37059700	1.19875300
H	2.36522000	0.37059500	-1.19875700
H	0.56100100	-0.74190100	-2.40564100
H	0.56100300	-0.74189900	2.40563900
H	-1.70054200	-0.92797300	-1.51064800
H	-1.70054100	-0.92797300	1.51064900
H	-0.92272000	1.61904100	0.00000000
H	-2.54029600	0.81289100	0.00000000

f 140 in clyclohexane (smd)

C	1.41455200	0.22006500	0.69227900
C	1.41455200	0.22006400	-0.69228100
C	0.33276600	-0.27149700	-1.46349800
C	0.33276600	-0.27149500	1.46349700
C	-0.97828000	-0.26771200	-1.06488000
C	-0.97828000	-0.26771200	1.06487900
C	-1.45550100	0.65223700	0.00000000
H	2.36168600	0.38810800	1.19519400
H	2.36168600	0.38810600	-1.19519600
H	0.57645700	-0.77207800	-2.39596000
H	0.57645700	-0.77207600	2.39595900
H	-1.70252900	-0.90093400	-1.56641400
H	-1.70252900	-0.90093400	1.56641300
H	-0.92173200	1.60419700	0.00000000
H	-2.53257500	0.79906900	0.00000000

*8.2. Isomerization between **14** and **15**. f (forward) and b (backward) indicate the sign of the reaction coordinate.*

b 120 in argon (smd)

C	1.11982300	1.28760300	0.08714500
C	-0.12310600	1.84899100	0.19883300
C	-1.35463800	1.12503700	0.08036500
C	-0.15136000	-0.86756200	0.35182200
C	1.27769500	-0.14830800	0.07061300
C	-1.35908400	-0.23375000	0.00005300
H	-0.19499900	2.92931300	0.24793100
H	1.21084500	-1.45341900	2.07411900
H	-2.28507900	1.66756400	-0.03617400
F	2.19860100	-0.59316600	-0.86886700
F	-2.42659600	-0.91097900	-0.45475500
H	1.98476000	1.91652000	-0.09304200
C	0.89155200	-1.05559500	1.12255200

b 240 in argon (smd)

C	1.13053600	1.30206300	0.14691000
C	-0.11027700	1.86173800	0.19092500
C	-1.34380300	1.12498700	0.02727400
C	-0.09641700	-0.84336100	0.28759000
C	1.24493800	-0.15267400	0.08225300
C	-1.33927200	-0.22744600	-0.03425500
H	-0.19290900	2.94038300	0.25753700
H	1.13036300	-1.45294700	2.12071100
H	-2.27585500	1.66138600	-0.10162200
F	2.15076700	-0.59282300	-0.88196600
F	-2.41533800	-0.93251200	-0.40735500
H	2.01657000	1.92322000	0.08509100
C	0.87621700	-1.07637500	1.14218500

b 360 in argon (smd)

C	1.14069100	1.30604900	0.21980600
C	-0.09622400	1.86889200	0.16708400
C	-1.32319800	1.12989800	-0.06207900
C	-0.08860900	-0.83415400	0.27611200
C	1.23912300	-0.15246900	0.10841100
C	-1.32550100	-0.22348300	-0.06764900
H	-0.18551600	2.94657200	0.24235500
H	1.11271900	-1.54343600	2.09440300
H	-2.25149400	1.66652600	-0.21460100
F	2.11449600	-0.56462400	-0.89860500
F	-2.41487300	-0.94917300	-0.34906000
H	2.03220200	1.92109600	0.25825000
C	0.86984800	-1.11725700	1.13368800

f 77 in argon (smd)

C	1.11820600	1.26461900	0.03029000
C	-0.14197500	1.83430900	0.20823200
C	-1.35569800	1.13760100	0.11678500
C	-0.30080900	-0.94048000	0.48520500
C	1.39194100	-0.11006300	0.06440700
C	-1.37309900	-0.24804300	0.02720200
H	-0.19456400	2.91609400	0.25632300
H	1.27987900	-1.46704800	1.94758800
H	-2.28654300	1.68821400	0.03997400
F	2.26239600	-0.58380500	-0.85948000
F	-2.43826000	-0.89413700	-0.49341100
H	1.92649800	1.90658300	-0.30783300
C	0.87894700	-1.02169600	1.04717000

f 153 in argon (smd)

C	1.11873000	1.25501800	0.00325800
C	-0.14854600	1.82810500	0.21180300
C	-1.35788800	1.14185800	0.13377800
C	-0.35220000	-0.96560300	0.53622900
C	1.42605500	-0.09733100	0.05652300
C	-1.38182700	-0.25530400	0.04280000
H	-0.19476000	2.91039300	0.25894800
H	1.29921300	-1.46998200	1.89713600
H	-2.28741100	1.69755600	0.07447200
F	2.29570800	-0.57855000	-0.85305900
F	-2.44266600	-0.88584700	-0.50839000
H	1.89721400	1.89893300	-0.39643600
C	0.86833900	-1.01138300	1.01701300

f 360 in argon (smd)

C	1.11747300	1.24553900	-0.02959800
C	-0.15456200	1.82091300	0.21339400
C	-1.36204100	1.14507200	0.15523500
C	-0.39770100	-0.98770100	0.58447600
C	1.45010500	-0.08834900	0.04330400
C	-1.39373900	-0.26239600	0.06248000
H	-0.19502900	2.90365400	0.26040500
H	1.31784900	-1.47674000	1.84448200
H	-2.28955200	1.70665300	0.11880700
F	2.33678800	-0.57219600	-0.84250600
F	-2.44698800	-0.87505000	-0.52358700
H	1.86585400	1.88985300	-0.48274200
C	0.85621900	-1.00373500	0.98729600

9.0. References

(1) Kozuch, S.; Martin, J. M. L. *Phys. Chem. Chem. Phys.* **2011**, *13*, 20104.

(2) Santra, N.; Sylvetsky, G.; Martin, J. M. L. *J. Phys. Chem. A* **2019**, *123*, 5129.

(3) Grimme, S.; Antony, J.; Ehrlich, S.; Krieg, H. *J. Chem. Phys.* **2010**, *132*, 154104.

(4) Grimme, S.; Ehrlich, S.; Goerigk, L. *J. Comput. Chem.* **2011**, *32*, 1456.

(5) Papajak, E.; Leverentz, H. R.; Zheng, J.; Truhlar, D. G. *J. Chem. Theory Comput.* **2009**, *5*, 1197.

[6] Dunning, Jr., T. H.; Peterson, K. A.; Wilson, A. K. *J. Chem. Phys.* **2001**, *114*, 9244.

(7) Prascher, B.; Woon, D. E.; Peterson, K. A.; Dunning, Jr., T. H. *Theor. Chem. Acc.* **2011**, *128*, 69.

(8) Peterson, K. A.; Woon, D. E.; Dunning, Jr., T. H. *J. Chem. Phys.* **1994**, *100*, 7410.

(9) Woon, D. E.; Dunning, Jr., T. H. *J. Phys. Chem.* **1994**, *101*, 8877.

[10] Feller, D.; Peterson, K. A.; Hill, J. G. *J. Phys. Chem.* **2011**, *135*, 044102.

(11) Adler, T. B.; Knizia, G.; Werner, H.-J. *J. Chem. Phys.* **2007**, *127*, 221106.

(12) Knizia, G.; Adler, T. B.; Werner, H.-J. *J. Chem. Phys.* **2009**, *130*, 054104.

(13) Barone, V. *J. Chem. Phys.* **2004**, *120*, 3059.

(14) Alecu, I. M.; Zheng, J.; Zhao, Y.; Truhlar, D. G. *J. Chem. Theory Comput.* **2010**, *6*, 2872–2887.

(15) Rubin, M. B. *J. Am. Chem. Soc.* **1981**, *103*, 7791.

(16) Marenich, A. V.; Cramer, C. J.; Truhlar, D. G. *J. Chem. Phys. B* **2009**, *113*, 4538.

(17) Marenich, A. V.; Cramer, C. J.; Truhlar, D. G. *J. Chem. Phys. B* **2009**, *113*, 6378.

(18) Bao, J. L.; Truhlar, D. G. *Chem. Soc. Rev.* **2017**, *46*, 7548.

(19) Ferro-Costas, D.; Truhlar, D. G.; Fernández-Ramos, A. *Pilgrim-version 2020.2;* University of Minneapolis: Minnesota, MN, and Universidade de Santiago de Compostela, Spain, 2020.

(20) Huisgen, R. *Angew. Chem. Int. Ed. Engl.* **1970**, *9*, 751–762.

7.1. Full Citation for Gaussian16 Software Package:

Frisch, M. J.; Trucks, G. W.; Schlegel, H. B.; Scuseria, G. E.; Robb, M. A.; Cheeseman, J. R.; Scalmani, G.; Barone, V.; Petersson, G. A.; Nakatsuji, H.; Li, X.; Caricato, M.; Marenich, A. V.; Bloino, J.; Janesko, B. G.; Gomperts, R.; Mennucci, B.; Hratchian, H. P.; Ortiz, J. V.; Izmaylov, A. F.; Sonnenberg, J. L.; Williams-Young, D.; Ding, F.; Lipparini, F.; Egidi, F.; Goings, J.; Peng, B.; Petrone, A.; Henderson, T.; Ranasinghe, D.; Zakrzewski, V. G.; Gao, J.; Rega, N.; Zheng, G.; Liang, W.; Hada, M.; Ehara, M.; Toyota, K.; Fukuda, R.; Hasegawa, J.; Ishida, M.; Nakajima, T.; Honda, Y.; Kitao, O.; Nakai, H.; Vreven, T.; Throssell, K.; Montgomery, J. A., Jr.; Peralta, J. E.; Ogliaro, F.; Bearpark, M. J.; Heyd, J. J.; Brothers, E. N.; Kudin, K. N.; Staroverov, V. N.; Keith, T. A.; Kobayashi, R.; Normand, J.; Raghavachari, K.; Rendell, A. P.; Burant, J. C.; Iyengar, S. S.; Tomasi, J.; Cossi, M.; Millam, J. M.; Klene, M.; Adamo, C.; Cammi, R.; Ochterski, J. W.; Martin, R. L.; Morokuma, K.; Farkas, O.; Foresman, J. B.; Fox, D. J. *Gaussian 16, Revision C.01*, Gaussian, Inc.: Wallingford CT, 2016.
